# Supplementary material for: Double mutation of open syntaxin and UNC-18 P334A leads to excitatory-inhibitory imbalance and impairs multiple aspects of C. elegans behavior
Source: bioRxiv. 2023 Nov 10:2023.08.18.553709. Originally published 2023 Aug 19. Preprint. [Version 2] doi: 10.1101/2023.08.18.553709 (PMC10462135; doi:10.1101/2023.08.18.553709)
Supplement: Supplement 1 [file media-1.pdf]

SUPPLEMENTARY INFO

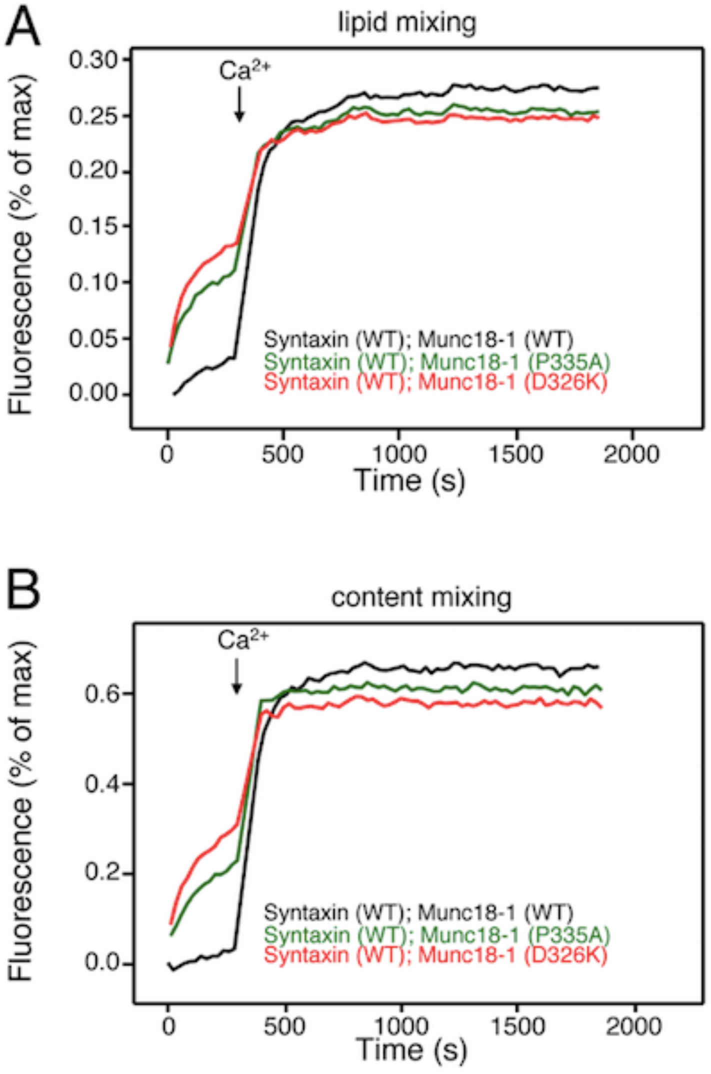

Supplementary Figure 1. Stimulatory effects of the P335A and D326K Munc18-1 mutations on Ca^{2+}-independent liposome fusion.

(A, B) Lipid mixing (A) between V-liposomes and S-liposomes containing WT syntaxin-1 was monitored from the fluorescence de-quenching of Marina Blue lipids, and content mixing (B) was monitored from the increase in the fluorescence signal of Cy5-streptavidin trapped in the V-liposomes caused by FRET with PhycoE-biotin trapped in the S-liposomes upon liposome fusion. Assays were performed in the presence of NSF, αSNAP, Munc13-1C and WT, P335A or D326K Munc18-1 as indicated by the color code. Experiments were started in the presence of 100 mM EGTA and 5 mM streptavidin, and Ca^{2+} (600 mM) was added at 300s.
